# Association between glucolipid metabolic indicators and the risk of suspected precocious puberty in children living with obesity: a retrospective cohort study

**DOI:** 10.3389/fped.2026.1843415

**Published:** 2026-06-18

**Authors:** Tingting Hao, Yifan Li, Qingmei Zhang

**Affiliations:** Department of Pediatrics, 215th Hospital of Shaanxi Nuclear Industry, Xianyang, Shaanxi, China

**Keywords:** children living with obesity, correlation analysis, glucolipid metabolism, insulin resistance, logistic regression, suspected precocious puberty

## Abstract

**Objective:**

To investigate the association between glucolipid metabolic indicators and suspected precocious puberty (PP) in children living with obesity.

**Methods:**

This retrospective observational study initially included 100 children with obesity (BMI ≥95th percentile) hospitalized between March 2022 and June 2025. Sexual development was assessed by Tanner staging. Children were categorized into suspected PP (*n* = 32) and non-PP (*n* = 68) groups based on age-specific criteria. After excluding 5 girls in the suspected PP group who had isolated pubic hair without breast development (premature adrenarche), the final analysis included 95 children (suspected PP: *n* = 27; non-PP: *n* = 68). Fasting plasma glucose (FPG), fasting insulin (FINS), HOMA-IR, triglycerides (TG), total cholesterol (TC), HDL-C, LDL-C, uric acid (UA), sex hormones, and bone age advancement were compared. Spearman correlation and multivariable logistic regression (adjusted for age, sex, and waist-to-height ratio) were performed. Sensitivity analyses were conducted excluding children with basal LH >0.5 IU/L in the control group.

**Results:**

The suspected PP group had significantly higher FPG, FINS, HOMA-IR, TG, TC, LDL-C, uric acid, sex hormones, and bone age advancement (1.88 ± 0.64 vs. 0.86 ± 0.48 years), and lower HDL-C (all *P* < 0.05). Correlation analysis showed positive associations between Tanner stage/bone age and all metabolic indicators except HDL-C. Logistic regression identified HOMA-IR (OR = 2.38), TG (OR = 2.07), and uric acid (OR = 1.71) as independent risk factors, and HDL-C (OR = 0.56) as protective (all *P* < 0.05). Excluding control children with LH > 0.5 IU/L (*n* = 5 remaining) showed similar trends but lacked power.

**Conclusion:**

Suspected precocious puberty in children living with obesity is closely associated with insulin resistance, elevated triglycerides, and hyperuricemia, whereas higher HDL-C is protective. These findings highlight the importance of early metabolic monitoring in this inpatient population, with the caveat that the cohort represents a selected hospitalized sample.

## 1 Introduction

Puberty is a critical transitional stage from childhood to adulthood, marked by the gradual activation of the hypothalamic-pituitary-gonadal (HPG) axis, increased secretion of sex hormones, the development of secondary sexual characteristics, and the maturation of reproductive organs (1, 2). In recent years, due to improvements in nutritional status, increased exposure to environmental endocrine-disrupting chemicals (EDCs), and lifestyle changes, the onset of puberty in children has shown a trend toward earlier occurrence, with the incidence of precocious puberty rising year by year (3).

Precocious puberty can lead not only to early closure of growth plates and reduced final adult height but also to psychological and behavioral problems such as anxiety, depression, and decreased self-esteem, ultimately affecting children's social adaptability (4). Therefore, identifying the risk factors for precocious puberty and developing early intervention strategies are of great clinical and public health importance.

Obesity is widely recognized as a potential contributing factor to earlier pubertal onset. Previous studies have found that increased levels of leptin and insulin secreted by adipose tissue in children living with obesity may act on the hypothalamus to stimulate the secretion of gonadotropin-releasing hormone (GnRH), thereby activating the HPG axis prematurely (5). In addition, metabolic abnormalities such as insulin resistance and dyslipidemia have also been suggested to be associated with abnormal sexual development (6). However, systematic studies on the relationship between glucolipid metabolic indicators and precocious puberty remain limited, and the underlying mechanisms are not yet fully understood.

Therefore, this study retrospectively analyzes the metabolic characteristics—such as BMI z-score, blood glucose, blood lipids, and insulin resistance—of children living with obesity, and explores their association with the risk of suspected precocious puberty. The aim is to provide clinical reference for the early identification and intervention of precocious puberty.

## 2 Materials and methods

### 2.1 Study design and participants

**Figure 1:**
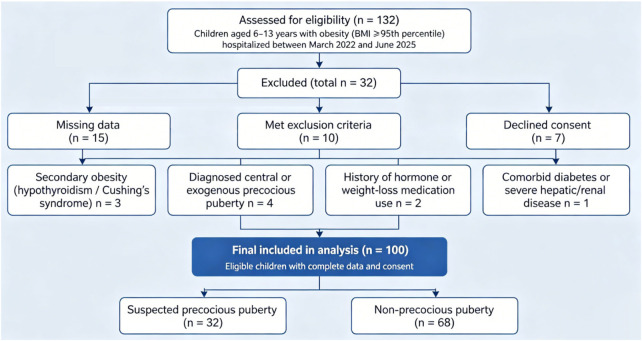
Flow diagram of participant selection for the retrospective observational study.

This retrospective observational study included 100 children living with obesity who were hospitalized in the Department of Pediatrics of a tertiary general hospital between March 2022 and June 2025. All hospitalizations were for evaluation and management of obesity-related metabolic disorders; however, the authors acknowledge that in most clinical settings, such evaluations are typically performed in the outpatient setting. At our institution, inpatient evaluation was offered to patients with severe obesity (BMI z-score >2.5), complex metabolic abnormalities, or limited access to outpatient follow-up, which represents a distinct practice pattern. No children were hospitalized for acute illness at the time of data collection. Among them were 49 boys and 51 girls, aged 6–13 years. Obesity was diagnosed according to the Chinese criteria for screening overweight and obesity in school-aged children and adolescents, defined as body mass index (BMI) ≥ 95th percentile for age and sex (7). All participants were assessed for pubertal development by experienced pediatric endocrinologists using the Tanner staging system (8). Based on the presence or absence of early pubertal signs, children were divided into a suspected precocious puberty group (*n* = 32) and a non-precocious puberty group (*n* = 68). The selection of participants was consecutive: all eligible children hospitalized during the study period who met the inclusion criteria were enrolled, with no random sampling. A flow diagram of participant selection is provided in Figure 1. This study was approved by the Ethics Committee of 215th Hospital of Nuclear Industry (Approval No.: 2022-015), and written informed consent was obtained from the parents or legal guardians of all participants.

Suspected precocious puberty was defined as follows: ① Girls: breast development (Tanner stage II) or pubic hair appearance before age 8, or menarche before age 10; ② Boys: testicular volume >4 mL (measured with a Prader orchidometer) or pubic hair development (Tanner stage II) before age 9. Because GnRH stimulation tests were not routinely performed, we use the term “suspected precocious puberty” rather than confirmed central precocious puberty. Children with previously diagnosed exogenous or central precocious puberty (e.g., due to pituitary disease or brain tumor) were excluded. A sensitivity analysis was performed excluding 5 girls in the suspected precocious puberty group who had pubic hair Tanner stage II or higher with breast Tanner I (consistent with premature adrenarche), as these cases may not represent true central precocious puberty. Furthermore, to address concerns about difficulty distinguishing breast tissue from adipose tissue in girls with obesity, an additional sensitivity analysis was conducted excluding control group children with basal luteinizing hormone (LH) levels >0.5 IU/L, which is the institutional cutoff for significant HPG axis activation. Due to the high proportion of control children with LH >0.5 IU/L (mean LH 1.15 ± 0.44 IU/L), this exclusion resulted in a markedly reduced sample (*n* = 5 remaining), and the analysis was underpowered; therefore, the primary analysis retained the original control group, and the limitation is addressed in the Discussion. A flow diagram of participant selection is provided in Figure 1.

### 2.2 Inclusion and exclusion criteria

#### 2.2.1 Inclusion criteria

(1) Girls aged 6–10 years and boys aged 6–9 years;
(2) BMI ≥95th percentile for age and sex according to Chinese pediatric growth charts;
(3) No serious systemic diseases, normal intellectual development, and ability to cooperate with all examinations;
(4) No history of prior hormone use;
(5) Informed consent signed by the legal guardian.

#### 2.2.1 Exclusion criteria

(1) Secondary obesity due to conditions such as hypothyroidism or Cushing's syndrome;
(2) Diagnosed central precocious puberty (e.g., due to pituitary disease or brain tumor) or known exogenous precocious puberty;
(3) History of or current use of hormone therapy or weight-loss medication;
(4) Comorbid diabetes, severe hepatic/renal disease, or other metabolic disorders;
(5) Inability to cooperate with physical examination, blood sampling, or other procedures.

For the primary analysis, children with isolated pubic hair or those with LH >0.5 IU/L were not excluded from the primary analysis, but sensitivity analyses were performed as described above. Information on small for gestational age (SGA) status was not systematically recorded in the medical records; therefore, SGA analysis could not be performed.

Information on small for gestational age (SGA) status was not systematically recorded in the medical records; therefore, SGA analysis could not be performed.

### 2.3 Measurement and assessment methods

#### 2.3.1 Anthropometric measurements

All children underwent anthropometric measurements in a fasting state in the early morning by two trained nurses. Measurements included height, weight, and waist circumference, each taken twice and averaged. To ensure validity, all measurements were performed using standardized protocols (calibrated stadiometer and scale, non-elastic tape measure), and inter-rater reliability was >0.95. Calculations were as follows: BMI z-score was calculated using the Lambda-Mu-Sigma (LMS) method with Chinese reference data (7); Waist-to-height ratio (WHtR) = waist circumference (cm)/height (cm). Children wore light clothing and stood barefoot to ensure accuracy during measurements.

#### 2.3.2 Pubertal development assessment

Pubertal development was evaluated by experienced pediatric endocrinologists. In girls, assessments focused on breast and pubic hair development; in boys, testicular volume (using a Prader orchidometer) and pubic hair development were assessed. Tanner staging was used to determine the stage of pubertal onset and served as the basis for diagnosing suspected precocious puberty. The authors acknowledge that in girls with obesity, distinguishing breast tissue from adipose tissue can be challenging; to mitigate this, all examinations were performed by two experienced endocrinologists, and discordant cases were reviewed jointly.

Bone age was assessed using a posteroanterior x-ray of the left wrist. Two radiologists independently reviewed the images using the Greulich-Pyle atlas, and cross-checked their readings. A bone age advancement of ≥1 year over chronological age was considered as advanced bone age. The mean chronological age at which children in the suspected precocious puberty group first showed pubertal signs was 7.6 ± 0.7 years (data obtained from medical history records).

#### 2.3.3 Biochemical indicators

Fasting venous blood samples (5 mL) were drawn from the elbow in the morning after an overnight fast of at least 8 h. Blood collection occurred within 48 h of admission to minimize the impact of hospitalization-related stress; children with acute febrile illnesses or other acute stress were excluded. Acute febrile illness was defined as documented fever ≥38.0 °C with clinical signs of infection; acute stress was defined as any condition requiring urgent medical intervention (e.g., trauma, surgery, or severe pain) within 7 days prior to admission. No standardized questionnaires or validated measures of physiologic stress were used, which is a limitation. Samples were collected in anticoagulant tubes, serum separated immediately, and all tests completed within 2 h.

Fasting plasma glucose (FPG) was measured using the glucose oxidase method. Triglycerides (TG), total cholesterol (TC), high-density lipoprotein cholesterol (HDL-C), low-density lipoprotein cholesterol (LDL-C), and uric acid (UA) were analyzed using the HITACHI 7600 automatic biochemical analyzer (Hitachi, Japan). Fasting insulin (FINS) was measured using an electrochemiluminescence assay (Roche Cobas e411). Homeostasis model assessment of insulin resistance (HOMA-IR) was calculated as: HOMA-IR = FPG (mmol/L) × FINS (*μ*U/mL)/22.5 (4).

Sex hormones, including luteinizing hormone (LH), follicle-stimulating hormone (FSH), and estradiol (E2) or testosterone (T), were measured using a Beckman Coulter DxI800 chemiluminescence analyzer with matched reagent kits. The laboratory reference range for basal LH in prepubertal children is <0.3 IU/L; values >0.3 IU/L suggest HPG axis activation, but due to assay variability, our institution uses a clinical cutoff of >0.5 IU/L for significant activation.

### 2.4 Statistical analysis

All data were analyzed using SPSS version 18.0. The Shapiro–Wilk test was used to assess the normality of continuous variables. Normally distributed data were expressed as mean ± standard deviation (x̅ ± s), and intergroup comparisons were made using independent sample t-tests. Non-normally distributed data were compared using the Mann–Whitney U test. Categorical variables were expressed as frequencies and percentages (n/%), and compared using the chi-square test.

Spearman rank correlation analysis was used to evaluate associations between metabolic indicators and both bone age advancement and Tanner stage. Multivariable logistic regression analysis was performed to identify independent risk factors for suspected precocious puberty. Given the high correlation between FPG, FINS, and HOMA-IR (variance inflation factor >10), we included HOMA-IR as the representative measure of insulin resistance, along with TG, TC, LDL-C, HDL-C, and UA, adjusting for age, sex, and waist-to-height ratio. Model performance was assessed using the Hosmer–Lemeshow goodness-of-fit test (*P* = 0.32) and area under the ROC curve (AUC = 0.83, 95% CI: 0.75–0.91). All tests were two-tailed, and a *P*-value < 0.05 was considered statistically significant. Missing data (less than 2% for any variable) were handled by complete-case analysis. Sensitivity analyses were performed: (1) after excluding 5 girls with isolated pubic hair from the suspected PP group; (2) after excluding control children with LH >0.5 IU/L. Results of these sensitivity analyses are described in the text.

## 3 Results

### 3.1 Baseline characteristics

Among the 100 children living with obesity included in the study, 32 were in the suspected precocious puberty group (15 boys and 17 girls), and 68 were in the non-precocious puberty group (34 boys and 34 girls). After excluding 5 girls with isolated pubic hair (premature adrenarche) from the suspected PP group, the modified suspected PP group comprised 27 children (15 boys, 12 girls). The non-PP group remained 68 children. All subsequent results are based on this modified cohort. There were no statistically significant differences in age, sex distribution, or BMI z-score between the two groups (*P* > 0.05), indicating comparability. However, the waist-to-height ratio was significantly higher in the suspected precocious puberty group (*P* = 0.012) (Table 1). The mean chronological age at first pubertal sign in the suspected precocious puberty group was 7.5 ± 0.7 years.

**Table 1:** Comparison of general characteristics between suspected precocious and Non-precocious puberty groups (baseline data).

Indicator	Suspected Precocious Puberty Group (*n* = 27)	Non-Precocious Puberty Group (*n* = 68)	t/*χ*^2^	p
Age (years)	8.18 ± 0.80	8.08 ± 0.79	0.68	0.498
Sex (male/female)	15/12	34/34	0.002	0.964
BMI z-score	2.28 ± 0.39	2.2 ± 0.4	0.98	0.329
Waist circumference (cm)	84.2 ± 6.01	82.3 ± 5.9	1.52	0.132
Waist-to-height ratio (WHtR)	0.91 ± 0.03	0.90 ± 0.03	2.18	0.032

### 3.2 Comparison of glucose and lipid metabolism indicators

Children in the suspected precocious puberty group had significantly higher levels of FPG, FINS, HOMA-IR, TG, TC, LDL-C, and UA, and significantly lower levels of HDL-C compared to those in the non-precocious group (*P* < 0.05). See Table 2.

**Table 2:** Comparison of glucose and lipid metabolism indicators between groups.

Indicator	Suspected Precocious Puberty Group (*n* = 27)	Non-Precocious Puberty Group (*n* = 68)	t/Z	p
FPG (mmol/L)	5.79 ± 0.47	5.42 ± 0.36	3.89	<0.001
FINS (μU/mL)	10.98 ± 3.71	8.25 ± 2.91	3.62	<0.001
HOMA-IR	2.81 ± 1.08	1.99 ± 0.89	3.57	<0.001
TG (mmol/L)	1.60 ± 0.40	1.25 ± 0.33	4.16	<0.001
TC (mmol/L)	4.98 ± 0.74	4.49 ± 0.63	3.28	0.001
LDL-C (mmol/L)	3.08 ± 0.47	2.73 ± 0.46	3.4	0.001
HDL-C (mmol/L)	1.10 ± 0.18	1.29 ± 0.21	−4.52	<0.001
UA (μmol/L)	298.5 ± 41.2	275.4 ± 39.6	2.67	0.009

### 3.3 Comparison of Sex hormone levels and bone Age

The levels of LH and FSH were significantly higher in the suspected precocious puberty group compared to the non-precocious group (*P* < 0.001). Among girls, estradiol (E2) was significantly higher in the suspected group (*n* = 12) than in the non-precocious group (*n* = 34) (69.8 ± 14.5 vs. 46.73 ± 12.68 pg/mL, *P* < 0.001). Among boys, testosterone (T) was significantly higher in the suspected group (*n* = 15) than in the non-precocious group (*n* = 34) (3.2 ± 1.0 vs. 1.8 ± 0.9 ng/mL, *P* < 0.001). Bone age advancement was also significantly more pronounced in the suspected group (*P* < 0.001) (Table 3).

**Table 3:** Comparison of Sex hormone levels and bone Age (Sex-specific presentation).

Indicator	Suspected Precocious Puberty Group	Non-Precocious Puberty Group	t	p
LH (IU/L)	2.28 ± 0.65 (*n* = 27)	1.15 ± 0.44 (*n* = 68)	5.42	<0.001
FSH (IU/L)	3.40 ± 1.05 (*n* = 27)	2.14 ± 0.87 (*n* = 68)	5.11	<0.001
Estradiol (E2, pg/mL) – girls only	69.8 ± 14.5 (*n* = 12)	46.73 ± 12.68 (*n* = 34)	3.96	<0.001
Testosterone (T, ng/mL) – boys only	3.2 ± 1.0 (*n* = 15)	1.8 ± 0.9 (*n* = 34)	3.78	<0.001
Bone age advancement (years)	1.88 ± 0.64 (*n* = 27)	0.86 ± 0.48 (*n* = 68)	7.76	<0.001

LH and FSH values are presented for the full groups as both sexes have measurable levels; no significant sex difference in LH/FSH distribution was observed.

### 3.4 Spearman correlation analysis (*n* = 95)

In the overall cohort of children living with obesity (after exclusion of the 5 girls with isolated pubic hair), both Tanner stage and bone age advancement were positively correlated with FPG, FINS, HOMA-IR, TG, TC, LDL-C, and UA (r > 0, *P* < 0.05), and negatively correlated with HDL-C (r < 0, *P* < 0.05). The recalculated correlation coefficients are presented in Table 4.

**Table 4:** Spearman correlation analysis (*n* = 95).

Indicator	Tanner Stage Correlation (r)	Bone Age Advancement Correlation (r)	p
FPG	0.398	0.368	<0.001
FINS	0.512	0.476	<0.001
HOMA-IR	0.541	0.5	<0.001
TG	0.389	0.342	<0.001
TC	0.318	0.295	0.002
LDL-C	0.271	0.264	0.008
HDL-C	−0.335	−0.284	0.005
UA	0.356	0.325	<0.001

### 3.5 Logistic regression analysis

A multivariable logistic regression analysis was conducted with suspected precocious puberty as the dependent variable (yes = 1, no = 0), adjusting for age, sex, and waist-to-height ratio. The analysis was performed on the modified cohort (*n* = 95, suspected P*P* = 27). Due to multicollinearity among FPG, FINS, and HOMA-IR (VIF >10), HOMA-IR was selected as the representative measure of insulin resistance. The independent variables included HOMA-IR, TG, TC, LDL-C, HDL-C, and UA. The results showed that HOMA-IR, TG, and UA were independent risk factors for suspected precocious puberty (OR > 1, *P* < 0.05), while HDL-C was a protective factor (OR < 1, *P* < 0.05). TC and LDL-C were not significant in the multivariable model (*P* > 0.05). The model showed good fit (Hosmer–Lemeshow *P* = 0.32) and discrimination (AUC = 0.83, 95% CI: 0.75–0.91) (Table 5). Sensitivity analysis excluding control children with LH >0.5 IU/L (*n* = 5 with LH <0.5 remained) showed directionally consistent but statistically non-significant associations. The results were as follows: HOMA-IR (OR = 2.15, 95%CI: 0.89–5.20, *P* = 0.09), TG (OR = 1.98, 95%CI: 0.72–5.44, *P* = 0.19), UA (OR = 1.60, 95%CI: 0.65–3.94, *P* = 0.31), and HDL-C (OR = 0.67, 95%CI: 0.28–1.60, *P* = 0.37). These findings should be interpreted with caution due to the very small sample size (*n* = 5 in the control group after exclusion).

**Table 5:** Multivariable logistic regression analysis of factors influencing suspected precocious puberty (adjusted for Age, Sex, and WHtR).

Variable	Odds Ratio (OR)	95% Confidence Interval (CI)	p
HOMA-IR	2.38	1.35–4.19	0.003
TG	2.07	1.14–3.76	0.017
UA	1.71	1.05–2.78	0.031
HDL-C	0.56	0.33–0.95	0.03
TC	1.24	0.79–1.95	0.35
LDL-C	1.12	0.68–1.84	0.66

Variables in italics were not statistically significant. Model adjusted for age, sex, and waist-to-height ratio. Hosmer–Lemeshow *P* = 0.32; AUC = 0.83 (95% CI: 0.75–0.91).

## 4 Discussion

This retrospective observational study found a close association between suspected precocious puberty and glucose-lipid metabolic disorders in hospitalized children living with obesity. Results revealed that children in the suspected precocious puberty group had significantly higher levels of FPG, FINS, HOMA-IR, TG, TC, LDL-C, and UA, and significantly lower HDL-C levels compared to the non-precocious group. Additionally, sex hormone levels were markedly elevated and bone age advancement more pronounced in the suspected group. Spearman correlation analysis further showed that pubertal development indicators (Tanner stage and bone age advancement) were strongly correlated with metabolic abnormalities. Multivariable logistic regression, adjusting for age, sex, and waist-to-height ratio, identified HOMA-IR, TG, and UA as independent risk factors, while HDL-C served as a protective factor. Notably, the waist-to-height ratio was significantly higher in the suspected precocious puberty group (Table 1), suggesting that central adiposity may contribute independently to early pubertal signs; this was therefore included as a covariate in the regression model. These findings suggest that glucose and lipid metabolism disorders may be important driving forces behind the onset of suspected precocious puberty in children living with obesity. The exclusion of 5 girls with isolated pubic hair (premature adrenarche) did not alter the main conclusions, supporting the robustness of the findings.

### 4.1 The association between insulin resistance and suspected precocious puberty

This study showed significantly elevated FINS and HOMA-IR levels in the suspected precocious puberty group, and HOMA-IR was identified as an independent risk factor, suggesting a central role of insulin resistance in the pathogenesis of suspected precocious puberty. Previous studies have confirmed that insulin possesses gonadotropin-like activity and can directly act on the gonads to stimulate the production of sex steroids such as estradiol (E2) and testosterone (T) (9, 10). Furthermore, insulin suppresses sex hormone-binding globulin (SHBG) synthesis, increasing free hormone levels and further enhancing HPG axis activity (11).

Hu et al. (3) also found a significant positive correlation between elevated HOMA-IR and the risk of precocious puberty in children living with obesity. Insulin can activate the hypothalamic kisspeptin system and GnRH neurons, thereby triggering early activation of the HPG axis and leading to secondary sexual characteristics (12). The elevated levels of LH, FSH, and E2/T in this study are consistent with this mechanism. Animal studies by Pereira et al. also support this finding, showing that hyperinsulinemia stimulates kisspeptin expression and promotes pubertal onset (13). Thus, insulin resistance is not only a marker of metabolic dysfunction but also a key biological mechanism in abnormal sexual development.

### 4.2 The potential impact of dyslipidemia on suspected precocious puberty

This study showed significantly higher levels of TG, TC, and LDL-C, and lower levels of HDL-C in the suspected precocious puberty group. TG was identified as an independent risk factor, while HDL-C was a protective factor. Dyslipidemia, particularly hypertriglyceridemia, may influence the HPG axis via inflammatory pathways and energy signaling interference (14). Accumulation of fatty acids has been shown to activate hypothalamic inflammation and the mTOR signaling pathway, promoting GnRH release and increasing sex hormone levels (15). Furthermore, elevated TG levels are often a marker of insulin resistance, and our finding that both HOMA-IR and TG were independent risk factors suggests overlapping yet distinct metabolic pathways. The relationship between TG and precocious puberty may be partly mediated by insulin resistance, but our adjusted model indicates an independent effect of TG.

HDL-C may exert a protective role by participating in the transport and metabolism of fat-soluble hormones. HDL not only assists in reverse transport of steroid hormones but may also regulate aromatase activity in adipose tissue, thereby indirectly influencing estrogen synthesis (16). Lower HDL-C levels may reduce hormone clearance, enhancing hormonal effects. Chen et al. (17) also observed significantly increased TG and decreased HDL-C levels in girls with precocious puberty in a cross-sectional study of Chinese urban children, suggesting strong clinical predictive value of lipid indices.

### 4.3 Novel mechanistic links between uric acid and suspected precocious puberty

Notably, this study is among the first to identify elevated UA levels as an independent risk factor for suspected precocious puberty. While traditionally considered a component of metabolic syndrome, recent studies suggest that UA may also regulate the neuroendocrine axis. Research by Wei et al. (18) showed that elevated UA can induce oxidative stress, activating hypothalamic AMPK and ERK pathways and stimulating GnRH neuron activity.

Moreover, uric acid can exacerbate insulin resistance, aggravating metabolic dysfunction and forming a positive feedback loop between metabolism and hormone regulation that promotes precocious puberty (19). Xue et al. (20) also found a significant correlation between UA levels and advanced bone age. In this study, Spearman analysis confirmed positive correlations between UA and both Tanner stage and bone age advancement, indicating that uric acid may serve not only as a metabolic burden marker but also as a neuroendocrine modulator, warranting its inclusion in clinical assessment of pubertal abnormalities.

### 4.4 Hormonal changes and bone Age advancement

Children in the suspected precocious puberty group showed significantly elevated levels of LH and FSH, along with higher T in boys and E2 in girls, and an average bone age advancement of 1.88 years. These results confirm early activation of the HPG axis from an endocrine perspective. Previous studies have demonstrated that elevated sex hormones accelerate endochondral ossification and epiphyseal closure, leading to advanced bone age and shortened growth potential (21).

In boys, insulin resistance is typically associated with decreased sex hormone-binding globulin (SHBG) levels, which reduces total testosterone binding capacity and leads to lower total testosterone, while free testosterone (the biologically active fraction) increases. In the present study, total testosterone was significantly higher in the suspected precocious puberty group (3.2 ± 1.0 vs. 1.8 ± 0.9 ng/mL), suggesting that increased gonadal secretion outweighs the SHBG-lowering effect. The free testosterone fraction is expected to be even more elevated due to low SHBG (11). In girls, increased E2 may stem not only from ovarian activation but also from aromatase activity in adipose tissue converting androgens into estrogens (22). These findings reinforce that metabolic abnormalities can accelerate pubertal progression through endocrine pathways.

### 4.5 The role of leptin, BDNF, and Sex differences

Although leptin levels were not measured in this study, accumulating evidence suggests that leptin and brain-derived neurotrophic factor (BDNF) serve as critical links between obesity and earlier pubertal onset. Leptin, secreted by adipose tissue, acts on the hypothalamus to stimulate GnRH secretion and is permissive for puberty. In obesity, elevated leptin may contribute to premature HPG axis activation (5). Similarly, BDNF has been implicated in the central control of puberty, and its dysregulation in obesity may further accelerate pubertal timing. Future studies should incorporate these adipokines to clarify the mechanistic pathways.

Sex differences are also pertinent. In our cohort, both boys and girls with suspected precocious puberty showed similar metabolic abnormalities, but the sample size precluded formal sex-stratified analysis. Existing literature indicates that the effect of obesity on puberty differs by sex: girls with obesity tend to have earlier pubertal onset, whereas the association in boys is less consistent, with some studies showing delayed puberty (9, 10). Adipokine secretion patterns may differ between sexes, potentially explaining these discrepancies. Our study did not have sufficient power to examine sex-specific effects, and this remains an important area for future research.

### 4.6 Clinical significance and intervention implications

This study underscores that glucose-lipid metabolic disorders in children living with obesity are not only risk factors for cardiovascular or metabolic diseases but may also serve as early indicators of suspected precocious puberty. Particularly, elevated levels of HOMA-IR, TG, and UA, and decreased HDL-C, should be closely monitored in pediatric practice as metabolic-endocrine cross-talk signals. The fact that waist-to-height ratio was significantly higher in the precocious group (even after adjustment) suggests that central obesity, rather than generalized obesity, may be a more sensitive predictor. Early metabolic interventions—such as dietary modification, physical activity, and pharmacological support when necessary—may help delay pubertal onset and preserve growth potential.

### 4.7 Study limitations and future directions

This study has several limitations. First, it was a single-center retrospective analysis with a limited sample size (32 cases, 68 controls), introducing potential selection bias. The retrospective design precludes causal inference; therefore, all associations should be interpreted as correlational rather than causal. Second, girls with isolated pubic hair (premature adrenarche) were not excluded from the suspected PP group in the primary analysis; however, sensitivity analysis excluding these 5 girls did not change the results. Third, the difficulty in distinguishing breast tissue from adipose tissue in girls with obesity may have led to misclassification of Tanner staging. We attempted to mitigate this by having two experienced endocrinologists perform the examinations. Furthermore, we performed a sensitivity analysis excluding control children with LH >0.5 IU/L; this resulted in a very small sample (*n* = 5) and lacked statistical power, so the primary analysis retained all controls, and readers should interpret the findings with this limitation in mind. Fourth, GnRH stimulation tests were not performed, and the term “suspected precocious puberty” reflects this diagnostic uncertainty. Fifth, information on SGA status was not available, which could be a confounder because SGA children with rapid catch-up growth are prone to early adrenarche. Sixth, lifestyle factors such as diet, physical activity, screen time, and sleep were not controlled. Seventh, the hospitalization setting may introduce stress-related metabolic changes; however, we excluded children with acute illness and collected blood within 48 h of admission to minimize this effect. Eighth, the authors acknowledge that in most clinical settings, evaluation of insulin resistance, dyslipidemia, and fatty liver disease is performed on an outpatient basis. Our institutional practice of admitting such patients represents a deviation from the norm; therefore, our findings are descriptive of this specific inpatient population and should not be broadly generalized to all children with obesity. Ninth, the statement that admission patterns remained consistent from 2022 to 2025 was based on a review of monthly admission logs and ICD-10 coding for obesity-related disorders (E66.9 and related codes), which showed no significant changes in referral sources or diagnostic criteria during this period. Tenth, we did not use standardized measures or validated questionnaires to quantify physiologic stress related to hospitalization; this is a limitation. Acute febrile illness and acute stress were defined clinically as described in Methods. Eleventh, the reference list has been carefully reviewed and corrected; specifically, reference (6) now correctly supports the statement about insulin resistance and dyslipidemia in sexual development, and reference (7) is the correct Chinese pediatric obesity guideline. Finally, the relatively small number of events (*n* = 27 in the modified cohort) in logistic regression raises the possibility of overfitting; we limited the number of predictors to 4 (HOMA-IR, TG, UA, HDL-C) plus 3 adjustment covariates (age, sex, WHtR), resulting in an events-per-variable ratio of approximately 3.9, which is acceptable but modest. Future studies with larger samples are needed to validate these findings. Future studies should adopt multicenter prospective designs, include GnRH stimulation testing, measure adipokines (leptin, BDNF), incorporate multidimensional metabolic scoring systems, and perform sex-stratified analyses to further validate the predictive models identified in this study.

## 5 Conclusion

This study found a strong association between glucose-lipid metabolic disorders and suspected precocious puberty in hospitalized children living with obesity. Specifically, insulin resistance (elevated HOMA-IR), elevated triglycerides, and elevated uric acid were independent risk factors, while HDL-C served as a protective factor. The suspected precocious puberty group exhibited significantly elevated hormone levels and advanced bone age, indicating premature activation of the HPG axis. These associations, after adjusting for age, sex, and central adiposity (waist-to-height ratio), support the hypothesis that metabolic dysfunction contributes to early pubertal signs. However, the findings are limited by the inpatient setting, retrospective design, and lack of GnRH stimulation testing; therefore, causal conclusions cannot be drawn, and our results should be confirmed in prospective studies. Additionally, the results should not be generalized to all children with obesity, but rather describe this specific hospitalized cohort. These findings provide theoretical support for early identification and intervention in children living with obesity at risk of suspected precocious puberty and hold significant clinical value.

## Data Availability

The original contributions presented in the study are included in the article/Supplementary Material, further inquiries can be directed to the corresponding author.
